# Annual abundance of common Kestrels (*Falco tinnunculus*) is negatively associated with second generation anticoagulant rodenticides

**DOI:** 10.1007/s10646-021-02374-w

**Published:** 2021-03-26

**Authors:** Staffan Roos, Steve T. Campbell, Gill Hartley, Richard F. Shore, Lee A. Walker, Jeremy D. Wilson

**Affiliations:** 1 RSPB Centre for Conservation Science, 2 Lochside View, EH12 9DH Edinburgh, UK; 2 Swedish Species Information Centre, Swedish University of Agricultural Sciences, Box 7007, 750 07 Uppsala, Sweden; 3 Science and Advice for Scottish Agriculture (SASA), Scottish Government, Roddinglaw Road, Edinburgh, EH12 9FJ UK; 4 UK Centre for Ecology & Hydrology, Lancaster Environment Centre, Library Avenue, Bailrigg, Lancaster LA1 4AP UK

**Keywords:** SGAR, Raptors, Poisoning, Sub-lethal effects, Birds of prey, Breeding Bird Survey

## Abstract

Rats and mice can damage food and agricultural products as well as transmit diseases, thereby requiring control of their numbers. Application of Second Generation Anticoagulant Rodenticides (SGARs) often reduces rodent numbers locally. However, predators eating rodents, including non-target species, that have consumed SGARs may be secondarily exposed and potentially lethally poisoned. Here we study whether SGARs may have contributed to the widespread population declines of a rodent-eating raptor, the Common Kestrel (*Falco tinnunculus*) in the UK. We show that 161 (66.8%) of the 241 Kestrels submitted for ecotoxicology tests between 1997 and 2012 had detectable levels of at least one SGAR in their livers. Adult Kestrels had significantly higher prevalence of SGARs than juveniles, suggesting accumulation of SGARs through time. The prevalence and concentrations of individual SGARs in Kestrels were significantly higher in England than in Scotland. SGAR prevalence in Kestrels were positively associated with some land cover types, primarily *arable cereals* and *broad-leaved woodland*, and negatively associated with mainly *mean elevation*, probably reflecting variation in SGAR usage across land cover types. By using volunteer-collected data on national Kestrel abundance 1997–2012, we show that there is a negative correlation between the Kestrel population index in a specific year and the concentration of bromadialone as well as the total SGAR concentration in the same year. Although correlative, this is the first study to provide evidence for a potential population-limiting effect of SGARs on a raptor.

## Introduction

Rodents can be a serious threat to human health through disease transmission, can damage food and agricultural products (Himsworth et al. 2013), and are predators of ground-nesting birds, especially on oceanic islands where they are invasive alien species and have caused many avian extinctions (Jones et al. 2011). Humans therefore often control rodent numbers, particularly of House Mice (*Mus musculus*) and Brown Rats (*Rattus norvegicus*). By using rodenticides, large-scale reduction and local extirpation of rats and mice can be accomplished for the benefit of human health, agriculture (Himsworth et al. 2013) and conservation of birds and other wildlife (e.g. Towns and Broome 2003).

In response to rodents evolving resistance to warfarin and other first generation anticoagulant rodenticides (Quy et al. 1992; Cowan et al. 1995), second generation anticoagulant rodenticides (SGARs) with higher toxicity were developed and these are now used globally to control rodent populations. They are dispensed in baits, and once consumed by a rodent, they interrupt the blood clotting mechanism by inhibiting the enzyme vitamin K epoxide reductase (Valchev et al. 2008). In most cases, the key target species are the Brown Rat and House Mouse, but especially in southern Europe other rodents (e.g. Water Voles *Arvicola amphibius*) can be target species (Coeurdassier et al. 2014; Martínez‐Padilla et al. 2017). However, *Microtus* voles and insectivores, who are not target species in the UK and many other countries, may also consume the baits (Brakes and Smith 2005; Dowding et al. 2010; Tosh et al. 2012).

Predators and scavengers that consume dead and dying rodents that have eaten rodenticides are also exposed to the active compounds. Previous studies have shown widespread exposure to SGARs in a diverse range of predators in Europe and North America (Newton et al. 1999, Thomas et al. 2011, Christensen et al. 2012; Coeurdassier et al. 2014; Sainsbury et al. 2018; Nakayama et al. 2019). For most predator species, few poisoned individuals are reported to the authorities, although it is possible that individuals exposed to a lethal dose of rodenticides die undetected. Thus, the true number of predators dying from secondary SGAR poisoning is unknown. The concentration of SGARs sufficient to be lethal for avian and mammalian predators is largely unknown (but see e.g. Erickson and Urban 2004; Thomas et al. 2011). There is within-species variation in the sensitivity to SGARs, particularly related to age and sex (e.g. Martínez‐Padilla et al. 2017), and sub-lethal effects such as weight loss, reduced body condition and immunosuppression are also reported (Serieys et al. 2015; Martínez‐Padilla et al. 2017).

The Common Kestrel (*Falco tinnunculus*; henceforth “Kestrel”) is a small falcon inhabiting open landscapes such as farmland, moorland and steppe. Small rodents dominate its diet, and in north-west Europe, voles of the genus *Microtus* are an especially important food source (Village 1990). The Kestrel has had volatile population dynamics since the 1940s. Once a very common raptor in Europe, organochlorine pesticides used in agriculture during the 1950s and 1960s caused direct mortality and reduced fecundity by egg-shell thinning in Kestrels and many other raptor species (Newton 1979). This led to a substantial population decline (Newton 1979, 2017). However, the population recovered strongly in the late 1960s and early 1970s (Village 1990; Woodward et al. 2018), following voluntary moratoria on the use of some of these pesticides (e.g. aldrin and dieldrin). After reaching a peak in abundance in 1974, the UK Kestrel population has declined significantly (i.e. −32% between 1995 and 2017; Harris et al. 2019). This decline is echoed across Europe (i.e. a significant −24% population decline between 1980 and 2016; Pan-European Common Bird Monitoring Scheme 2018). The reasons for these recent population declines are unknown, but hypotheses include intensification of agricultural practices (Village 1990, Chamberlain et al. 2000; Butet et al. 2010), habitat loss through afforestation (Village 1990), locally increased levels of intra-guild predation from Northern Goshawks (*Accipiter gentilis*; Petty et al. 2003), increased levels of competition for cavity nest sites by increasing populations of e.g. Barn Owls (*Tyto alba*) and Jackdaws (*Corvus monedula*; Charter et al. 2010), a general decline in the Kestrel’s main prey (i.e. *Microtus* voles; Cornulier et al. 2013), and increased use of SGARs resulting in higher mortality of Kestrels (e.g. Christensen et al. 2012).

At least two circumstances support the hypothesis that SGARs could have a population limiting effect on Kestrels. Firstly, several studies have found SGAR residues in non-target rodents, such as mice of the genus *Apodemus*, Bank Voles *Myodes glareolus* and Field Voles *Microtus agrestis* (Brakes and Smith 2005; Tosh et al. 2012), all of which are included in the diet of Kestrels and would increase the probability that Kestrels are exposed to SGARs. A Spanish study confirmed this by showing that Kestrel nestlings hatched in an agricultural area where the local authorities provided rodenticides to farmers to combat vole outbreaks had significantly higher levels of that SGAR (bromadiolone) in their blood than Kestrel nestlings hatched in an area where no SGAR was provided to the farmers (Martínez‐Padilla et al. 2017). Secondly, in the agricultural habitats widely occupied by Kestrels, many farms and rural businesses use SGARs to control rodent numbers (Hughes et al. 2013). For example, in the late 1990s, 91% of gamekeepers in Great Britain reported the use of rodenticides, 95% of which were anticoagulants (McDonald and Harris 2000). In England, SGAR usage in industrial, housing and agricultural settings increased by 11% between 1997 and 2001 (Dawson and Garthwaite 2003). Similarly, in Scotland the percentage of arable farms using SGARs increased from 80.1% to 96.9% between 2000 and 2010 (Hughes et al. 2013). During the years with comparable data, SGAR usage was higher in England than Scotland (Dawson and Garthwaite 2003). However, the total amount of rodenticide used on arable farms declined by 57% in Scotland between 2014 and 2018, probably because the introduction of an industry-led stewardship scheme in 2015 (CRRU 2015) resulted in increased adherence to best practice procedures (Reay et al. 2019).

Here we examine the potential contribution of SGARs to the population decline of Kestrels in the UK by combining data on SGAR exposure in Kestrels and the species’ abundance, measured through the annual long-term BTO/JNCC/RSPB Breeding Bird Survey (BBS) conducted in April to June each year since 1994. We first describe how two different analytical methods to estimate SGAR concentrations (the early and less sensitive method of high-performance liquid chromatography, HPLC, and the current and more sensitive method of liquid chromatography mass spectrometry, LCMS) could be combined for temporal analyses. Secondly, we explore whether SGAR exposure varies according to Kestrel age classes and sex. Thirdly, we test whether SGAR concentrations in Kestrels vary across the UK. Fourthly, we test whether there are associations between SGAR concentrations in Kestrels and the land cover in the county they were found. Finally, we test whether there are associations between temporal changes in SGAR concentrations and population changes in Kestrels. We hypothesised that the Kestrel population index in a specific year would be negatively associated with the prevalence and concentration of SGARs found in Kestrels in the period since the previous Breeding Bird Survey (i.e. the period between 1 July in year *t* and 30 June in year *t* + 1).

## Methods

### Kestrel population data

Since 1994, the Breeding Bird Survey (BBS) has monitored the abundance of breeding birds in the UK (Harris et al. 2019). The BBS is a line-transect survey based on randomly located 1-km squares. Squares are chosen through stratified random sampling, with more squares in areas with more potential volunteer surveyors. The difference in sampling densities is considered when calculating trends. Observers make two early-morning visits to squares during the April - June survey period, recording all adult birds encountered while walking two 1-km transects across their square. Species-specific population trends are then estimated using the maximum count of individuals over the two visits. Only squares that have been surveyed in at least two years are included in the trend analyses. Population changes are estimated using a log-linear model with Poisson error terms. Counts are modelled as a function of year and site, weighted to account for differences in sampling effort across the UK, with standard errors adjusted for overdispersion. For many common species, trends are produced both for the whole of the UK as well as for the four constituent countries in the UK (i.e. England, Scotland, Wales and Northern Ireland). Details of the BBS methodology, including trend estimations, are given in Harris et al. (2019).

Kestrel BBS trends are produced for the whole of the UK as well as for England and Scotland. All these trends show significant population declines between 1995 and 2017, but the declines are more severe in Scotland (−59%) than in England (−17%) and UK nationwide (−32%). For the analyses in this study, we used the annual UK Kestrel population index values (Woodward et al. 2018; available online at www.bto.org/birdtrends). Because the span of years with data on SGARs in Kestrels was shorter than the duration of the whole BBS trend, we only used BBS data for the years 1997–2012. We set the population index in 1997 to 100. An increase in Kestrel abundance above the 1997 value had an index value of >100 and a decline had an index value <100.

### SGARs in Kestrel tissues

Members of the public in the UK have submitted dead Kestrels to governmental and other organisations for ecotoxicology tests since 1992. For the purpose of this study, we used the estimates of the liver concentration of SGARs in 241 Kestrels (unit: μg SGAR/g wet liver weight) found dead and submitted between the years 1997 and 2011. We obtained the data from the Predatory Bird Monitoring Scheme (PBMS) at the UK Centre for Ecology and Hydrology and from the Science & Advice for Scottish Agriculture (SASA). Data from the PBMS were mainly from Kestrels in England (N = 201), but a few were from Scotland (N = 9) and Wales (N = 1). For four Kestrels analysed by the PBMS, provenance was not known. The data from SASA included 26 Kestrels, all found in Scotland. For certain analyses, some individuals were excluded, because variables such as provenance, sex or age class (juvenile or adult) were unknown. Although there are no published inter-calibration data between the two laboratories, the same analytical protocol was used in both. In addition, there was no significant difference in the probability of detecting at least one SGAR compound in the livers of Kestrels found in Scotland but analysed by the two different laboratories (N = 35, of which both PBMS and SASA had used the historical analytical method HPLC for three individuals; binomial GLM, F_1,33_ = 0.673, *p* = 0.418). Similarly, there was no significant difference in the total SGAR concentration in the livers of Kestrels found in Scotland but analysed by the two different laboratories (Welch two sample *t*test, *t* = 0.369, df = 9.028, *p* = 0.721). This suggests that the data from the two laboratories are comparable.

We considered data for three SGARs (brodifacoum, difenacoum and bromadialone) licensed for use in the UK. We did not analyse the levels of flocoumafen because it only occurred in one Kestrel. Details of the laboratory methods used to determine liver SGAR concentrations using high-performance liquid chromatography (HPLC) and liquid chromatography mass spectrometry (LCMS) can be found in, for example, Shore et al. (2003) and Walker et al. (2017), respectively.

A potential problem for this study was that it covered a period when the analytical methods changed. The early method to determine SGAR concentrations (HPLC) is less sensitive (i.e. had a higher minimum concentration at which it could be detected) than the current method (LCMS) (Dowding et al. 2010; Sainsbury et al. 2018). In our data, the laboratory at PBMS switched from HPLC to LCMS in 2006, whereas the laboratory at SASA switched in 2003. Previous studies have shown that it might be necessary to apply the historical (i.e. HPLC) levels of detection to the newer data (i.e. LCMS) when comparing temporal changes in SGAR prevalence and concentration (e.g. Sainsbury et al. 2018). We explored the differences between HPLC and LCMS as a preliminary step in our statistical analyses (see below).

### Data analysis

All statistical analyses were done in R (R Core Team 2017) and the geographical analyses in ArcGIS (ESRI 2011). Sample sizes for all analyses are in the Results and associated tables. The number of analysed Kestrels per year and county is in the Supporting Information (Table SI 1).

Throughout this paper, we modelled exposure in five ways: (i) probability of detecting the SGARs brodifacoum, difenacoum and bromadiolone, (ii) probability of detecting at least one SGAR (hereafter: “any SGAR”), (iii) the number of SGARs detected, (iv) the concentration of individual SGARs, and (v) the total concentration of all SGARs. Henceforth, we use the word *prevalence* to describe analyses related to (i)-(iii), and *concentration* to describe analyses related to (iv) and (v). In analyses regarding the number of SGARs, we used models with a Poisson error term, and we checked for model overdispersion using the R package “aods3” (Lesnoff and Lancelot 2018). All models were slightly underdispersed (dispersion factor: 0.782–0.925), which we regarded as acceptable to proceed with the analyses using Poisson error terms. To explore which variables were associated with variation in concentration levels, we only included Kestrels with detectable SGAR residues of interest for that specific analysis in (iv) and (v). We transformed the concentration values using the R package “rcompanion” (Mangiafico 2020) to achieve normal distributions. This package identifies the power transformation that makes the data fit the normal distribution as closely as possible by using iterative Shapiro–Wilk tests (Mangiafico 2020). In all cases, data became normally distributed following the transformations (Shapiro-Wilk’s test, *p* ≥ 0.093). Following the transformation, we checked the concentration data for heteroscedasticity using the Breush-Pagan test (Breusch and Pagan 1979) by applying the Non-constant Variance Score (NCV) test in the R program “car” (Fox and Weisberg 2019). In all cases, the data was not heteroscedastic (*p* ≥ 0.137).

#### Comparison of SGAR prevalence and concentrations in Kestrels based on HPLC and LCMS

We compared prevalence of the individual SGARs, any SGAR and the number of SGAR compounds detected between the samples of Kestrels tested using the historical HPLC and newer LCMS techniques. We also compared the mean (±1 SE) concentration of each SGAR and the total concentration of all SGARs in Kestrels tested by HPLC and LCMS techniques. For the tests of prevalence and the number of SGARs we used χ^2^-tests. For the tests of concentration, we used Welch two sample *t*-tests. The results of these comparisons revealed that the rate of detection of individual SGAR compounds as well as the number of SGARs detected differed significantly between the two methods (see Results). We therefore applied a level of detection (LoD) to the newer LCMS data that made it comparable to the historical HPLC data. Based on our results, we did this by reclassifying all SGAR estimates of <0.025 μg/ g wet liver weight from the LCMS data to 0 (for a similar approach, see Dowding et al. 2010; Sainsbury et al. 2018).

#### Age and sex-related differences in SGAR prevalence and concentrations in Kestrels

Only Kestrels tested for SGARs by the PBMS were aged and sexed. For this subset of individuals, where the sample size varied depending on the response variable (see Table 2), we tested whether the different measures of SGAR exposure (see above) differed between age class (juvenile and adult) and sex (male and female). For the SGAR estimates based on LCMS, we used estimates adjusted to the historical HPLC LoD (see above). We fitted models with the relevant SGAR measure as response variable and *age* and *sex* as explanatory variables and considered the interaction term *age* × *sex* to check whether effects of *age* and *sex* were additive or interacted. Specifically, we fitted generalized linear mixed models (GLMMs) with a binomial error term and logit link for response variables (i) and (ii), and with a Poisson error term and log link for response variable (iii). For response variables (iv) and (v), we fitted linear mixed models (LMMs) with the transformed concentration value as response variable. Because of the non-random sample of Kestrels across counties, we fitted *county* as random terms in all these models.

#### Land cover associations with SGAR concentrations in Kestrels

We did not know the exact location where each Kestrel was found. Thus, it was not possible to conduct analyses of habitat associations of discovery locations. However, we knew in which county each Kestrel was found. We therefore calculated county-specific mean SGAR prevalence and concentration in Kestrels. To examine differences in SGAR prevalence and concentrations between Scotland and England, we used χ^2^-tests and Welch two sample *t*-tests, respectively.

For each county, we calculated the mean elevation in ArcMap (ESRI 2011) using a Digital Elevation Model (DEM) with 50 m grid resolution (Ordnance Survey 2019). We also calculated the proportion of different land cover types in each county, using the digital Land Cover Map 2000 (LCM2000; Centre for Ecology and Hydrology 2000), which includes 26 different land cover categories. These were further reduced by pooling similar categories; “dwarf shrub heath” and “open dwarf shrub heath” was pooled into a new category labelled *Heath;* “Continuous urban” and “Suburban/rural developed” was pooled into *Urban and semi-urban;* and “Supra-littoral rock”, “Supra-littoral sediment”, “Littoral rock”, “Littoral sediment” and “Saltmarsh” were pooled into *Coastal*. We also eliminated habitat types that were rare (i.e. had a mean cover of less than 1%). To further reduce the number of environmental variables to take forward to the statistical analyses, we selected land cover types and elevation variables using Variation Inflation Factors (VIF). We excluded those factors with a VIF > 5. Ultimately, we used 10 variables (*broad-leaved woodland*, *coniferous woodland*, *arable cereals*, *improved grassland*, *neutral grassland*, *calcareous grassland*, *set-aside grassland*, *coastal*, *urban and semi-urban*, and *mean elevation*) in the statistical analyses. Despite using VIF as means to reduce the number of correlated variables, some relatively strong pairwise correlations remained. Specifically, *coniferous woodland* was positively correlated with *mean elevation* (r = 0.76), *arable cereals* was positively correlated with *set-aside grassland* (r = 0.72) and *arable cereals* was negatively correlated with *mean elevation* (r = −0.73). All other pairwise correlations had an |r | -value of less than 0.60.

To model associations between prevalence of SGARs and land cover categories and elevation, we used Generalized Linear Mixed Models (GLMM) with either a binomial error structure and a logit link (response variables (i) and (ii)), or a Poisson error structure and a log link response variable (iii). When modelling associations between SGAR concentrations in Kestrels (response variables (iv) and (v)) and land cover categories, we used Linear Mixed Models (LMMs) with normal errors. Because of the non-random sample of Kestrels across counties, we fitted *county* as random terms in these models. We used an information-theoretic approach to model selection (Burnham and Anderson 2002) by fitting a full model with all explanatory variables. We then ran all possible model permutations and ranked them using the Akaike Information Criterion corrected for small sample sizes (AICc; Akaike 1973; Burnham and Anderson 2002). The Akaike weight (ω_i_) of each model was calculated within the top set of models with a Δ-AICc ≤ 2 units, as well as the relative importance of each variable from within the top set of models (i.e. ∑ω_i_). Multi-model inference was used to determine the averaged effect size (β coefficient) of each variable across the top set of models (Burnham and Anderson 2002). Variables were standardized to have a mean = 0 and SD = 1 prior to analysis. To evaluate model performance, we calculated the conditional R^2^, which is the variance explained by the model, including fixed and random effects, for all models within the top set of models. We report mean, minimum and maximum R^2^-values of each model in the top set of models. Model fitting and multi-model inference was done using the R package MuMIn (Barton 2016).

#### Temporal changes in SGAR concentrations in Kestrels

Because we predicted that the Kestrel population index in one year would be negatively associated with the amount of SGARs used in the period since the previous Breeding Bird Survey (April-June), we assumed that each “year” started on the 1^st^ of July. Thus, Kestrels found between 1^st^ of July in year *t* and 30^th^ of June in year *t* + 1 were used to calculate annual SGAR means for year *t* + 1. For the first year of our study (1997), only Kestrels found between 1 January and 30^th^ of June 1997 were available (N = 7). For the last year of our study, which was the last year Kestrels were tested for SGARs (2011), 12 were found after 1^st^ of July and thus contributed to the 2012 annual mean.

We modelled temporal changes in the prevalence of SGARs using Generalized Linear Mixed Models (GLMMs) with a binomial error structure and a logit link (response variables (i) and (ii)) and with a Poisson error structure and a log link (response variable (iii)). To model temporal changes in concentrations of SGARs in Kestrels (response variables (iv) and (v)), we used Linear Mixed Models (LMMs). For the LMMs, we only included Kestrels with detectable levels of the SGAR of interest. For these models, we used the continuous variable *year* as the explanatory variable, and we specified *county* as a random effect to control for the non-random sample of Kestrels across counties.

#### Associations between SGARs and Kestrel abundance

The UK Kestrel population has undergone a significant population decline between 1995 and 2017 (Harris et al. 2019). To reduce the risk of identifying spurious associations between variables that change simultaneously but independently, we first transformed the data on Kestrel population index to achieve normal distribution and checked the data for heteroscedasticity. We then detrended the time series, using linear detrending in the R package “pracma” (Borchers 2019). The differences between the unadjusted and the detrended time series are shown in the Supporting Information (Fig. S1). We then fitted univariate linear regressions with the detrended Kestrel population index as the continuous response variable, and a measure of SGAR as the only explanatory variable. The measures of SGAR that we used, one at a time, were the continuous variables annual mean proportion of Kestrels with (a) presence of each individual SGAR, (b) presence of any SGAR, (c) the annual mean number of SGARs detected in each Kestrel, (d) the annual mean concentration of each individual SGAR, and (e) the annual mean total concentration of all SGARs. The mean values for (d) and (e) were from Kestrels with detectable SGAR levels only. Because the accuracy of the annual mean SGAR estimates was based on a variable number of individual Kestrels that have been tested for SGARs each year (range: 5–40 for the years 1997–2012), we weighted the analyses with the proportion of Kestrels tested in year *t* divided by the total number of Kestrels tested across all years (i.e. N_*t*_ /N_Tot_, meaning that higher weight was given to years with a higher number of Kestrels tested).

## Results

### The number of Kestrels found in different counties

In total, 241 Kestrels found dead in the UK were tested for SGARs between 1997 and 2012, using the year limits we set. This included only 35 from Scotland. Overall, Kestrels were found in 48 different counties (13 Scottish counties, 34 English counties and one Welsh county; Table SI 1). The number of Kestrels from the different counties varied greatly; from one Kestrel from 15 different counties to 38 Kestrels from one county (Cambridgeshire; Table SI 1).

#### Comparisons of SGAR prevalence and concentration in Kestrels based on HPLC and LCMS

Of the 241 Kestrels tested, 161 (66.8%) had detectable levels of at least one SGAR. Of these, 73 had residues of one SGAR, 59 had residues of two and 29 had residues of three. The two most frequent SGARs were difenacoum (120 Kestrels) and bromadiolone (N = 116). Brodifacoum was found in 41 Kestrels. Only one bird contained detectable levels of flocoumafen.

There was no difference in the proportion of Kestrels analysed with HPLC (65.4% of 100) and LCMS (69.3% of 88) that had detectable levels of at least one SGAR (*p* = 0.627; Table 1). However, for individual SGARs, there were important differences in detectability. Using HPLC, 11.1%, 48.4% and 38.6% of Kestrels had detectable levels of brodifacoum, difenacoum and bromadiolone, respectively (Table 1). In contrast, LCMS detected the same SGARs in 27.3%, 52.3% and 64.8% of the birds. For brodifacoum and bromadiolone, these differences were highly significant (i.e. *p* ≤ 0.002; Table 1). The number of detected SGARs was significantly different between the two methods (χ^2^ = 25.288, df = 3, *p* < 0.001). On average, Kestrels tested by HPLC had residues of 0.98 ± 0.07 SGAR compounds, whereas those tested by LCMS had 1.46 ± 0.12 SGAR compounds. Specifically, Kestrels tested with HPLC had only one SGAR more often than expected by chance (58 vs. 46 expected), whereas those tested with LCMS had one SGAR less often than expected by chance (15 vs. 27 expected). Even more striking was how rarely HPLC detected three SGARs in a Kestrel (i.e. 8 vs. 18 expected by chance), and how often LCMS detected three SGARs (i.e. 21 vs. 11 expected by chance).

**Table 1:** Comparison of the prevalence and concentration of Second Generation Anticoagulant Rodenticide (SGAR) compounds in the livers of 241 Kestrels collected in England (N = 205), Wales (N = 1) and Scotland (N = 35) between 1997 and 2011

SGAR compound	Method	Number (%) of Kestrels with detectable SGAR residues	χ^2^-test statistics, prevalence	Mean ± SE concentration (μg/g wet liver weight)	*t*-test statistics, concentration
Brodifacoum	HPLC	17 (11.1%)	**χ**^**2**^ = **9.223, df** = **1**, ***p*** = **0.002**	0.116 ± 0.033	***t*** = **2.759**, **df** = **38.753**, ***p*** = **0.009**
	LCMS	24 (27.3%)		0.155 ± 0.077	
Difenacoum	HPLC	74 (48.4%)	χ^2^ = 0.203, df = 1, *p* = 0.653	0.118 ± 0.024	***t*** = **2.683, df** = **92.190**, ***p*** = **0.009**
	LCMS	46 (52.3%)		0.061 ± 0.016	
Bromadialone	HPLC	59 (38.6%)	**χ**^**2**^ = **14.342, df** = **1,** ***p*** < **0.001**	0.236 ± 0.035	***t*** = **2.547**, **df** = **106.020**, ***p*** = **0.012**
	LCMS	57 (64.8%)		0.177 ± 0.033	
Total SGAR	HPLC	100 (65.4%)	χ^2^ = 0.236, df = 1, *p* = 0.627	0.246 ± 0.031	*t* = 0.495, df = 108.600, *p* = 0.621
	LCMS	61 (69.3%)		0.272 ± 0.049	

Significant differences are highlighted in bold. The analyses for the early part of the study (1997–2005 for Kestrels analysed by the Predatory Bird Monitoring Scheme and 1997–2002 for Kestrels analysed by Science and Advice for Scottish Agriculture) used high performance liquid chromatography (HPLC; N = 153) and the analyses for the later part of the study used liquid chromatography mass spectrometry (LCMS; N = 88). The LCMS is a more sensitive method and has a lower level of detection. The mean concentrations presented are based on the unadjusted SGAR values for Kestrels with detected SGAR. The *t*-tests used the transformed values of concentration. For other results in this study, the LCMS concentrations were adjusted for limits of detection to be comparable with the HPLC concentration (see Methods and Results). Significant results are highlighted in bold.

**Fig. 1:**
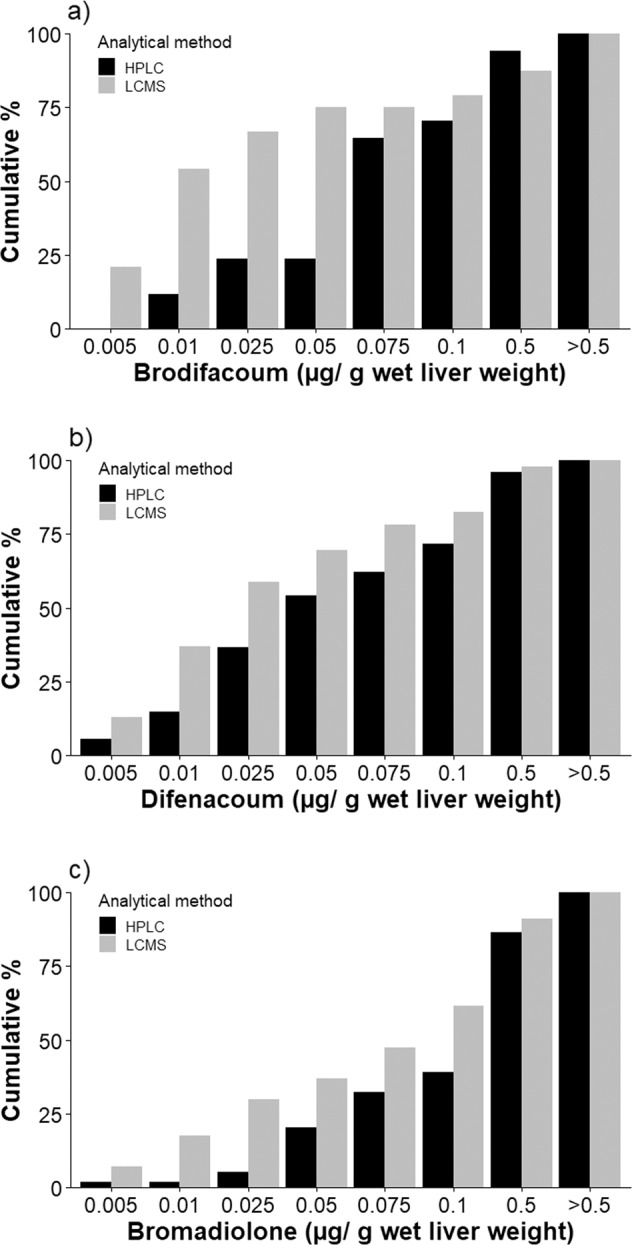
Frequency distribution of (**a**) brodifacoum, (**b**) difenacoum and (**c**) bromadiolone liver concentrations (μg/g wet liver weight) in Kestrels detected by the early and less sensitive method of high-performance liquid chromatography, HPLC, and the current and more sensitive method of liquid chromatography mass spectrometry, LCMS

There were few cases where HPLC detected brodifacoum and bromadiolone below 0.025 μg/g wet liver weight, whereas with LCMS, 66.7% and 29.8% of the individuals had brodifacoum and bromadiolone concentrations below this value (Fig. 1). Although there was no significant difference in the detection rate of difenacoum using the two analytical methods (Table 1), there were fewer Kestrels with difenacoum concentrations below 0.025 μg/g wet liver weight in the HPLC data (36.5%) than in the LCMS data (58.7%).

For the subset of birds with detected SGAR compounds, LCMS-tested birds had significantly higher concentration of brodifacoum than HPLC-tested birds (*p* = 0.009; Table 1), whereas for difenacoum and bromadiolone HPLC-tested birds had significantly higher concentrations (*p* ≤ 0.012; Table 1). There was no difference between the two methods in the total SGAR concentration (*p* = 0.621; Table 1).

Because of the difference between HPLC and LCMS in detecting low concentrations of SGARs, we adjusted all SGAR measurements of <0.025 μg/g wet liver weight from the LCMS data to 0 (see Methods). The remainder of this paper uses these adjusted values. Hence, the number of birds with detectable levels of the SGARs is smaller than when the unadjusted values are used.

#### Age and sex-related differences in SGAR prevalence and concentrations

For individuals where age and sex were known, *age* was, in general, more strongly associated with SGAR prevalence than was *sex*. The interaction term was not significant (*p* ≥ 0.123; Table 2), and *sex* was not significantly associated with any measure of SGAR prevalence (*p* ≥ 0.212; Table 2). Specifically, adult Kestrels were significantly more likely to have detectable residues of brodifacoum, bromadiolone and presence of at least one SGAR compound than juvenile Kestrels (*p* ≤ 0.034; Table 2). For difenacoum, this difference was marginally non-significant (*p* = 0.063; Table 2). In addition, adult Kestrels had residues of significantly more SGAR compounds than juveniles did (mean adults: 1.346 ± 0.096; mean juveniles: 0.752 ± 0.080; *p* < 0.001; Table 2). Specifically, there were fewer adults, but more juveniles, than expected by chance with no SGARs (i.e. 14 vs. 28 expected adults and 55 vs. 41 expected juveniles with no SGARs). Also, there were more adults (28 vs. 17 expected) and fewer juveniles (14 vs. 25 expected) with two SGAR compounds.

**Table 2:** Associations between *Age* and *Sex* and the prevalence and concentration Second Generation Anticoagulant Rodenticide (SGAR) compounds in the livers of Kestrels collected in the UK between 1997 and 2012

SGAR compound	N	Age	Sex	Interaction term
Brodifacoum, presence	183	−1.001 ± 0.472, χ^2^ = 4.502, ***p*** = **0.034**	0.289 ± 0.466, χ^2^ = 0.386, *p* = 0.534	χ^2^ = 0.009, *p* = 0.924
Brodifacoum, conc.	24	−0.111 ± 0.048, χ^2^ = 5.463, ***p*** = **0.019**	−0.024 ± 0.051, χ^2^ = 0.215, *p* = 0.643	χ^2^ = 0.089, *p* = 0.765
Difenacoum, presence	183	−0.607 ± 0.331, χ^2^ = 3.457, *p* = 0.063	0.399 ± 0.331, χ^2^ = 1.458, *p* = 0.227	χ^2^ = 1.988, *p* = 0.159
Difenacoum, conc.	79	0.060 ± 0.026, χ^2^ = 5.579, ***p*** = **0.018**	0.083 ± 0.026, χ^2^ = 10.180, ***p*** = **0.001**	χ^2^ = 1.659, *p* = 0.198
Bromadiolone, presence	183	−1.251 ± 0.331, χ^2^ = 14.322, ***p*** < **0.001**	−0.139 ± 0.326, χ^2^ = 0.183, *p* = 0.669	χ^2^ = 1.532, *p* = 0.216
Bromadiolone, conc.	86	0.020 ± 0.012, χ^2^ = 2.926, *p* = 0.087	0.010 ± 0.012, χ^2^ = 0.682, *p* = 0.409	χ^2^ = 0.601, *p* = 0.438
Any SGAR, presence	183	−1.439 ± 0.379, χ^2^ = 14.509, ***p*** < **0.001**	−0.038 ± 0.349, χ^2^ = 0.012, *p* = 0.914	χ^2^ = 0.806, *p* = 0.369
Total SGAR, conc.	123	0.007 ± 0.015, χ^2^ = 0.217, *p* = 0.641	0.028 ± 0.015, χ^2^ = 3.211, *p* = 0.073	χ^2^ = 0.006, *p* = 0.937
Number of SGARs	183	−0.524 ± 0.151, χ^2^ = 12.041, ***p*** < **0.001**	0.074 ± 0.150, χ^2^ = 0.244, *p* = 0.621	χ^2^ = 1.597, *p* = 0.206

Values are parameter estimates ± SE (reference level for *Age* = “Adult” and reference level for *Sex* = “Female”) from GLMMs with binomial error terms when modelling presence of SGARs and GLMMs with Poisson error terms when modelling the number of SGARs. The parameter estimates for concentrations of SGARs (only including birds with detectable levels of the SGAR and using the transformed values to achieve normal distribution) are from LMMs. In all models *Year* and *County* were included as random terms to control for non-random sample of birds across years and counties. The level of detection (LoD) from the modern and more sensitive liquid chromatography mass spectrometry (LCMS) technique was adjusted so that SGAR values of <0.025 μg/g wet liver weight were treated as 0. Only Kestrels with detectable levels of the individual SGARs are included in the tests of concentration. Untransformed means (±SE) are presented in Table SI 2. Significant results are highlighted in bold. The non-significant interaction terms were removed from the final models, which only included the variables *Age* and *Sex*.

In general, there were no associations between SGAR concentration measures and *age* and *sex* (Table 2). The exceptions were that juvenile Kestrels had significantly higher concentrations of difenacoum than adults (*p* = 0.018) and that male Kestrels had significantly higher concentrations of difenacoum than females (*p* = 0.001).

#### Differences in SGAR prevalence and concentration between England and Scotland

The concentration of difenacoum, bromadialone and total SGARs did not differ between Kestrels found in England and Scotland (*t*-tests, *t* ≤ 2.133, *p* ≥ 0.17; Table SI 2). It was not possible to test for differences in brodifacoum, because no Kestrels found in Scotland had detectable levels of this SGAR. However, the proportion of Kestrels with detectable levels of at least one SGAR was significantly higher in England than in Scotland (χ^2^ = 22.079, df = 1, *p* < 0.0001; Table SI 2). In addition, the number of SGAR compounds detected in each Kestrel was higher for English than Scottish Kestrels (χ^2^ = 26.409, df = 3, *p* < 0.0001; Table SI 2). These significant differences were largely because there were many more Kestrels in Scotland without any SGAR residues than expected by chance (27 vs. 14 expected), and fewer Kestrels in Scotland with two and three SGARs than expected by chance (in both cases 0 vs. 7 and 2 expected, respectively).

#### Prevalence and concentration of SGARs in relation to land cover

**Fig. 2:**
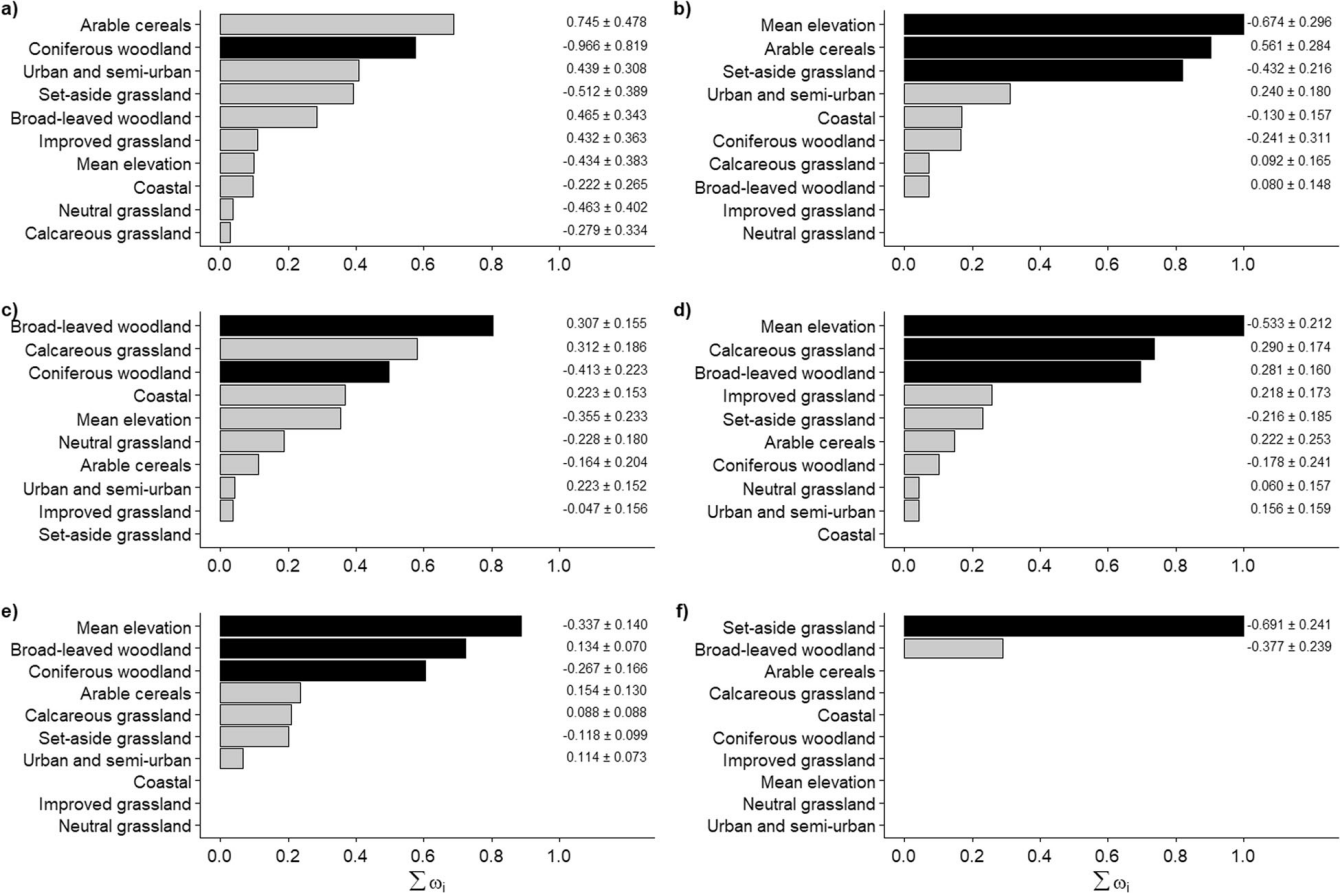
Relative importance of environmental variables explaining variation in the prevalence of (**a**) brodifacoum, (**b**) difenacoum, (**c**) bromadiolone, (**d**) presence of any SGAR and (**e**) the number of SGAR compounds in Kestrel livers. In (**f**) the, relative importance of environmental variables explaining variation in the concentration of brodifacoum. Variables are ranked in order of the sum of their Akaike weights (Σω_i_) within the top set of models, i.e. models with Δ-AICc ≤ 2. Black bars indicate those variables that were retained in the best single approximating model (i.e. the model with the lowest AICc-value) and grey bars indicate variables included in all other models within the top set. Variables without bars were not included in any model within the top set. Notation to the right of the bars indicates the strength of the slopes for each standardized variable

A set of competing models with county-specific environmental explanatory variables was produced for each measure of SGAR prevalence and concentration. For all measures of prevalence, several competing models had a Δ-AICc ≤ 2. In general, the variance explained by top set models, measured by the conditional R^2^, was low. In most cases, a few environmental variables explained most of the variation in SGAR prevalence (Fig. 2). Specifically, brodifacoum presence increased with the proportion of *arable cereals* but decreased with the proportion of *coniferous woodland* (mean R^2^ across models with Δ-AICc < 2: 0.095, range 0.016–0.350; Fig. 2a). Difenacoum presence declined at higher *mean elevation* and with higher proportion of *set-aside grassland* but increased with higher proportion of *arable cereals* (mean R^2^ = 0.171, range 0.144–0.198; Fig. 2b). Bromadiolone was positively associated with *broad-leaved woodland* and *calcareous grassland*, but negatively associated with c*oniferous woodland* (mean R^2^ = 0.090, range 0.072–0.118; Fig. 2c). The presence of any SGAR was negatively associated with *mean elevation*, but positively associated with *broad-leaved woodland* and c*alcareous grassland* (mean R^2^ = 0.119, range 0.0106–0.131; Fig. 2d). Finally, the number of SGAR compounds was negatively associated with *mean elevation* and *coniferous woodland*, but positively associated with *broad-leaved woodland* (mean R^2^ = 0.110, range 0.080–0.135; Fig. 2e).

Almost all SGAR concentration measures had no strong association with any environmental variables. For difenacoum, bromadiolone and total SGAR concentration, the null model (i.e. model with intercept only) had the best fit to the data, and all other competing models had a Δ-AIC ≫ 2. However, brodifacoum concentration was negatively associated with *set-aside grassland* (mean R^2^ = 0.305, range 0.266–0.344; Fig. 2f).

#### Temporal trends in SGAR prevalence and concentrations

There was a significant decline in the proportion of Kestrels with detectable levels of difenacoum between 1997 and 2012 (*p* = 0.009; Table 3a), but no significant trends for the prevalence of other SGARs, presence of any SGAR or for the number of SGARs detected (Table 3a).

**Table 3:** In (a) estimates from univariate Generalized Linear Mixed Models showing the associations between year and prevalence of individual SGAR compounds, presence of any SGAR and the number of SGARs in Kestrel livers. In (b) estimates from Linear Mixed Models showing the association between concentration (μg/g wet liver weight) of individual SGAR compounds and the total concentration of all SGARs. In (b), only Kestrels with detectable levels of each SGAR were included in the analyses. We set each year to start 1 July.

Response variable		N	Estimate ± SE	DF	χ^2^	*p*
(a) *Prevalence*						
Brodifacoum		241	0.029 ± 0.056	1	0.271	0.602
Difenacoum		241	−0.104 ± 0.040	1	6.849	**0.009**
Bromadiolone		241	0.048 ± 0.036	1	1.745	0.187
Presence of any SGAR		241	−0.039 ± 0.037	1	1.105	0.293
Number of SGARs		241	−0.009 ± 0.019	1	0.211	0.646
(b) *Concentration*						
Brodifacoum	Year	25	−0.048 ± 0.020	1	5.603	**0.017**
	Year^2^		0.003 ± 0.001	1	7.879	**0.005**
Difenacoum		93	0.001 ± 0.001	1	0.353	0.552
Bromadiolone		100	0.000 ± 0.001	1	0.124	0.725
Total SGAR		144	0.004 ± 0.003	1	2.850	0.091

Significant results are highlighted in bold.

There was a significant non-linear increase in the concentration of brodifacoum in Kestrels over the course of the study (Table 3b), but no significant trends for other compounds or the total concentration of all SGARs (Table 3b).

#### Associations between SGARs and population changes in Kestrels

**Fig. 3:**
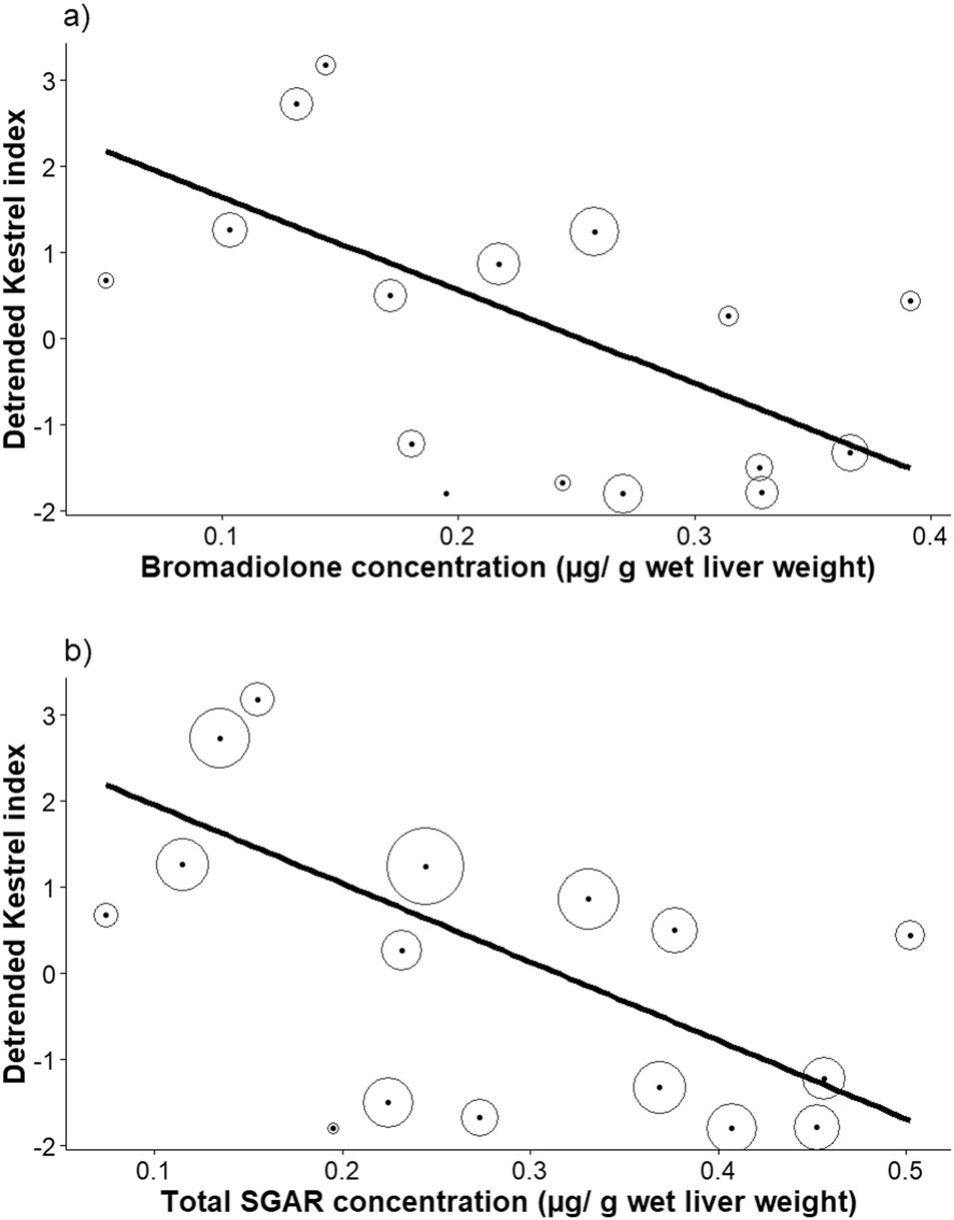
The relationship between the detrended annual UK Kestrel index (from the Breeding Bird Survey; Harris et al. 2019; see Methods for details of the detrending process) and the mean annual concentration (μg/g wet liver weight) of (**a**) bromadiolone, and (**b**) the total SGAR concentration. The filled points show the mean annual concentration, and the black solid lines show best linear fit to the data (R^2^ = 0.372 and 0.442 in figure (**a**) and (**b**), respectively). The analyses were weighted by the proportion Kestrels in each of the total sample size. The annual weighting factor is indicated by the size of the open circles

There were no significant associations between the detrended UK Kestrel population index and any measure of SGAR prevalence (Table 4a). However, the index was inversely associated with bromadiolone concentration as well as the total concentration of SGAR (*p* = 0.012 and *p* = 0.005, respectively; Table 4b and Fig. 3). There were no significant associations between the index and the concentrations of brodifacoum and difenacoum (Table 4b).

**Table 4:** Estimates from univariate linear regressions showing the associations between the UK population index of Kestrel and (a) the prevalence and (b) the concentration (μg/g wet weight in Kestrel livers) of the three most commonly used SGARs and the total concentration of all SGARs. In (b), only Kestrels with detectable levels of each SGAR contributed to the annual mean values.

Explanatory variable	Estimate ± SE	DF	F	*p*
*(*a*) Prevalence*				
Brodifacoum	5.311 ± 6.179	1, 14	0.739	0.405
Difenacoum	0.933 ± 2.650	1, 14	0.124	0.730
Bromadialone	−4.281 ± 3.515	1, 14	1.483	0.243
Presence of any SGAR	0.576 ± 3.172	1, 14	0.033	0.859
Number of SGARs	−0.199 ± 2.008	1, 14	0.010	0.922
(b) *Concentration*				
Brodifacoum	−1.505 ± 1.429	1, 9	1.110	0.320
Difenacoum	−3.953 ± 4.481	1, 13	0.778	0.394
Bromadialone	−10.795 ± 3.752	1, 14	8.278	**0.012**
Total SGAR	−9.109 ± 2.735	1, 14	11.085	**0.005**

Significant results are highlighted in bold.

## Discussion

We found SGAR residues in 161 (66.8%) of the 241 Kestrels that had been collected between 1997 and 2012, which is a higher prevalence than for some other raptors and owls analysed in the UK (19.2% of Tawny Owls (*Strix aluco*; Walker et al. 2008) and 38% of Common Buzzards (*Buteo buteo*; Shore et al. 2006), whose diet can be dominated by voles in certain vole peak years (Francksen et al. 2017)). Only Barn Owls, a generalist predator of small mammals, and Red Kites (*Milvus milvus*), which often scavenge dead rats, had prevalence of SGAR residues above those found in Kestrels (i.e. 87 and 100%, respectively; Walker et al. 2016, Walker et al. 2017; Shore et al. 2019). However, the high prevalence of SGARs in Kestrels in our study is lower than reported by Christensen et al. (2012) from Denmark, where 89% of Kestrels had residues of at least one anticoagulant rodenticide (including one first generation anticoagulant; coumatetralyl). In Denmark, ten other raptor and owl species also had very high prevalence of anticoagulant rodenticide residues (i.e. 84–100%; Christensen et al. 2012). The high prevalence of SGARs in Kestrels in both the UK and Denmark suggests that the main prey of Kestrels (i.e. voles and other small rodents) often consume SGAR baits (e.g. Brakes and Smith 2005; Tosh et al. 2012), and that Kestrels in turn are exposed to these toxins.

The improvements in analytical methods, which increased the sensitivity to detect low concentrations of SGARs, meant that we had to adjust the SGAR estimates from the current and sensitive laboratory method LCMS to be comparable to the estimates from the historic and less sensitive HPLC in order to make use of all the data. Our comparison of the two methods suggests that HPLC severely underestimates low-level SGAR exposure rate in Kestrels, and previous studies have shown similar differences between HPLC and LCMS for Barn Owl, Polecat (*Mustela putorius*) and Hedgehog (*Erinaceus europaeus*) (Dowding et al. 2010; Walker et al. 2012; Sainsbury et al. 2018). After these adjustments, our analyses showed that the prevalence of difenacoum declined between 1997 and 2012, whereas the concentrations of brodifacoum in Kestrel livers increased significantly during the same period (Table 3). All other measurements of SGAR exposure remained stable (Table 3). More interestingly, in years with high liver concentration of bromadiolone and a high total liver concentration of SGARs in Kestrels, the Kestrel population index was significantly lower (Table 4, Fig. 3), suggesting that SGARs may be a contributory factor in the overall significant population decline of Kestrels in the UK (i.e. −32% between 1995 and 2017; Harris et al. 2019). We are unaware of other studies linking anticoagulant rodenticides to population change of predators at a national scale. Our study, albeit correlation-based, therefore contributes important findings that could shed light on the significant population decline of Kestrels in the UK and elsewhere in Europe. However, corroborative studies from other parts of Europe, as well as experimental studies are needed to test this hypothesis in detail.

With few exceptions (e.g. Wardlaw et al. 2016; Reay et al. 2019), there are no available data on the annual usage of SGARs in the UK or the density of baits applied in different regions and habitats. Additionally, the proportion of prey individuals of target and non-target species that have consumed SGARs in an area is typically unknown (but see e.g. Brakes and Smith 2005), which makes it difficult to assess fine-scale spatial secondary SGAR exposure in predators. However, we have shown that there was much higher exposure rate in England than in Scotland. The higher proportion of built-up environment (e.g. urban and semi-urban settings as well as industrial estates; 11.1%) in England compared to Scotland (2.6%; Rae 2017), as well as the higher proportion of arable land in England (e.g. 43.1% arable land) compared to Scotland (10.1% arable land; Rae 2017) may contribute to explaining higher SGAR levels in Kestrels in England. It is also possible that rodenticide usage is higher in England than in Scotland, as supported by higher prevalence of SGAR residues in Barn Owls in England than in Scotland (Shore et al. 2015). Overall, it is probable that SGARs applied near domestic, industrial and farm buildings are a likely source of the SGARs that were detected in the Kestrels. Previous studies have suggested that users of SGARs had low awareness of the risk of secondary poisoning of non-target species (Tosh et al. 2011), and hence there may be a need of better education as well as statutory monitoring and regulation of how these highly toxic rodenticides are applied and used (Hughes et al. 2013). However, the recent introduction of the industry-led stewardship scheme (CRRU 2015) reassuringly appears to have resulted in, at least in Scotland, a decline in the usage of SGARs on farms and in the majority of both farmers and professional pest controllers now state that they comply with all elements of the best practice guidelines (Reay et al. 2019). It is also possible that the widespread use of SGARs near Pheasant (*Phasanius colchius*) release pens and feeding stations (McDonald and Harris 2000) may have contributed to the higher prevalence of SGARs in Kestrels in England than in Scotland, especially since the densities of Pheasants and Red‐legged Partridge (*Alectoris rufa*) released for shooting purposes are much higher in England than in Scotland (Balmer et al. 2013; Pringle et al. 2019). It is known that non-target rodents, such as Wood Mouse (*Apodemus sylvaticus*), Bank Vole and Field Vole consume rodenticides at Pheasant feeders (Brakes and Smith 2005).

Our results showed associations between some land cover classes and the prevalence of SGAR compounds in Kestrels (Fig. 2). However, the deviance explained by the top models with Δ-AICc < 2 was generally low, so we avoid making strong inference from these associations. One potential reason for the low deviance explained may be that a small percentage of UK Kestrels are partial migrants (Village 1990), and hence may have consumed prey with SGAR away from the place where it was found dead. Nevertheless, the prevalence of brodifacoum and difenacoum were positively related to the percentage of *arable cereals*, corroborating that SGAR usage is high on arable farms (Christensen et al. 2012; Hughes et al. 2013; Wardlaw et al. 2016). In line with this, the prevalence of difenacoum, presence of any SGAR and the number of SGAR compounds were negatively related to the *mean elevation*. Because *mean elevation* and *arable cereals* was strongly negatively correlated (r = −0.73), this gives some support to high usage of SGARs on arable farms. Similarly, the negative association between *coniferous woodland* and presence of brodifacoum, bromadiolone and the number of SGAR compounds may be an artefact of the strong positive correlation between *mean elevation* and *coniferous woodland* (r = 0.76). The associations between *calcareous grassland* and bromadiolone as well as the presence of any SGAR, and the negative association between *set-aside grassland* and the presence of difenacoum as well as the concentration of brodifacoum are difficult to interpret, because both grassland types occur in low percentage in all counties (mean area covered 5.3% and 1.9%, respectively). We are not aware of any land-use activities linked to these grassland types that would affect SGAR load. However, the positive associations between *broad-leaved woodland* and presence of bromadiolone, any SGAR and the number of SGAR compounds are intriguing. *Broad-leaved woodland* was not strongly correlated with other variables (all pairwise correlations: r ≥ 0.58; highest with *urban and semi-urban*), suggesting that there might be higher usage of SGARs in areas with high percentage cover of *broad-leaved woodland*. The high usage of SGARs near Pheasant release pens (McDonald and Harris 2000), which in England often are situated in broad-leaved woodlands (Sage et al. 2020), raises the question of whether the management of Pheasant releases could be a contributing factor to the positive associations between several SGAR prevalence measures and broad-leaved woodland (Fig. 2).

Despite the widespread and commonplace exposure of SGARs to Kestrels, none of the 241 Kestrels were found to have internal haemorrhaging unrelated to signs of trauma (i.e. suggestive of a lethal dose of SGARs; Walker et al. 2012). In fact, the concentration of the different SGARs that constitutes a lethal dose for Kestrels is unknown. Newton et al. (1999) suggested that liver SGAR residues in excess of 0.1–0.2 μg/g wet liver weight were “of concern” for Barn Owls, and residues >1 μg/g wet liver weight are considered “very high”. In our study, 64 (26.6%) and nine (3.7%) of the 241 Kestrels had a total liver concentration of SGARs above 0.2 and 1.0 μg/g wet liver weight, respectively. These birds may have been at risk of dying as a direct result of their high SGAR levels. However, it is also possible that non-lethal SGAR concentrations may affect Kestrel behaviour in other ways. For example, Kestrels with non-lethal SGAR concentrations may have lower body condition, be less agile, less vigilant or have a reduced immune system, making them more likely to be taken by predators, die in collisions with vehicles or contract disease (e.g. Riley et al. 2007; Martínez‐Padilla et al. 2017). It is unknown whether Kestrels that died from collisions with vehicles or were taken by other predators and later submitted for ecotoxicology tests were a representative sample of all Kestrels that die each year. Nevertheless, SGARs may limit Kestrel numbers in other ways than direct mortality via lethal internal haemorrhaging, or via sub-lethal physiological effects that increase the risk of being killed. For example, we found significantly higher prevalence of SGARs in adult compared to juvenile Kestrels (Table 2), suggesting accumulation of SGARs over time. Because many Kestrels do not start breeding until their second year (Village 1990), there is a risk that two years of accumulation of SGARs may lead to low recruitment into the breeding population. In addition, brodifacoum may have prolonged effects that increase the toxicity of subsequent exposures of the same or other anticoagulant rodenticides (Rattner et al. 2020). A high proportion of Kestrels in our study had residues of multiple SGARs (i.e. 36.5%), suggesting that repeat exposure to SGARs is common and therefore may increase risk of direct or indirect mortality, especially of adult Kestrels. Another way in which SGARs may limit Kestrel numbers is via reduced nestling survival. A recent study from Spain showed that Kestrel nestlings exposed to low levels of bromadiolone had on average 6.7% lower body mass and body condition than Kestrels without traces of bromadiolone (Martínez‐Padilla et al. 2017). Because body mass and body condition are strong predictors of future survival rate in many raptors, including Kestrels (Korpimäki and Rita 1996; McDonald et al. 2005; Colchero et al. 2017), the results of the study by Martínez‐Padilla et al. (2017) suggested that sub-lethal effects of SGARs on Kestrel nestlings may impair juvenile recruitment into the population. Experimental field studies are needed to test whether this may result in lower population size locally and regionally, and to what extent immigration from other areas may compensate for lower local recruitment. Finally, SGARs may also affect Kestrel numbers negatively by reducing the numbers of rodent prey following rodenticide campaigns. Thus, instead of a direct toxic effect, increased local SGAR usage causing reduced prey availability may lead some Kestrels to emigrate from an area and for those remaining to have reduced survival and reproductive rates.

Other predatory species that show similar or higher levels of exposure as Kestrels, such as Red Kites and Polecats, have recently increased in numbers and dramatically expanded their geographical range in the UK (Balmer et al. 2013; Sainsbury et al. 2018). This is most likely because these populations are expanding into previously unoccupied areas with low intra-specific competition over food and nest sites, which may outweigh any negative impact of SGARs.

### Limitations of our study

There are few sources of independent data on the amount of SGARs used in the UK (but see Reay et al. 2019 for estimates for SGAR usage on arable farms in Scotland), and the monitoring of concentrations in the tissues of dead Kestrels submitted for testing may not be a random sample of the Kestrel population. For example, the voluntary approach of submitting dead raptors, mainly by the public, may incur unknown biases, such as higher reporting rate from areas with high human population density. It is also possible that birds killed by collisions with windows and cars are more frequently submitted than birds that died in more natural circumstances away from human settlements. Indeed, the post-mortem examinations suggested most birds submitted for analyses have ultimately died from road traffic collisions and other trauma, or from starvation (Walker et al. 2014). It is unknown whether consumption of SGARs may lead to sub-lethal behavioural changes that make raptors more vulnerable to these causes of death. In addition, the number of birds submitted varies between months and years, and it is known that the concentrations of SGARs in some predators varies between seasons (e.g. Shore et al. 2003). To ensure an equal temporal spread of birds tested within a year, the Predatory Bird Monitoring Scheme at the Centre for Ecology and Hydrology only analyse a random subset of birds submitted from England each month. For Scotland, all 35 Kestrels submitted between 1997 and 2012 were analysed for SGARs.

## Conclusion

We have shown a widespread exposure to SGARs in UK Kestrels. There were significant positive associations between SGAR exposure and the proportion of land covered by *arable cereals* and *broad-leaved woodland*, probably reflecting land covers where SGAR usages is highest. We have also found a negative association between two measures of SGAR exposure (i.e. concentration levels of bromadiolone and the total SGAR concentration in Kestrels) and the population index of Kestrels. This suggests that it is possible that SGARs may have contributed to the significant population declines of Kestrels in the UK and elsewhere in Europe (Pan-European Common Bird Monitoring Scheme 2018; Harris et al. 2019). Because of the widespread Kestrel population declines across Europe, our study suggests that further work is urgently needed to examine pathways of secondary SGAR poisoning in Kestrels and other raptors. Furthermore, experimental work examining age- and sex-specific sensitivity to SGARs as well as breeding productivity and survival rates in relation to SGAR exposure would be welcome. In a UK perspective, further studies are needed to understand the reasons for the steeper Kestrel population declines in Scotland than in England. Our study does not provide evidence that SGARs are contributing disproportionately to the population decline of Kestrels in Scotland compared to England, because the SGAR prevalence in kestrel livers was lower in Scotland than in England.

## Supplementary information

Supplementary Information

## Data Availability

Data will be made available by SR upon reasonable request.

## Code availability

Code will be made available by SR upon reasonable request.
