# Supplementary material for: Annual abundance of common Kestrels (Falco tinnunculus) is negatively associated with second generation anticoagulant rodenticides
Source: Ecotoxicology. 2021 Mar 26;30(4):560–74. doi: 10.1007/s10646-021-02374-w (PMC8060177; doi:10.1007/s10646-021-02374-w)
Supplement: Supplementary file 1 — Supplementary Information [file 10646_2021_2374_MOESM1_ESM.docx]

# Supplementary Information

This Supplementary Information is linked to the paper "Annual abundance of Common Kestrels (*Falco tinnunculus*) is negatively associated with Second Generation Anticoagulant Rodenticides" by Roos et al., which was submitted to Ecotoxicology on 11^th^ of October 2020, and following comments from two reviewers, resubmitted on 31^st^ of January 2021.

**Authors**:

Staffan Roos^1, 2^*, Steve T. Campbell^3^, Gill Hartley^3†^, Richard F. Shore^4‡^, Lee A. Walker^4^, & Jeremy D. Wilson^1^

**Affiliations**:

^1^ RSPB Centre for Conservation Science, 2 Lochside View, EH12 9DH Edinburgh, UK

^2^ Swedish Species Information Centre, Swedish University of Agricultural Sciences, Box 7007, 750 07 Uppsala, Sweden

^3^ Science and Advice for Scottish Agriculture (SASA), Scottish Government, Roddinglaw Road, Edinburgh EH12 9FJ, UK

^4^ UK Centre for Ecology & Hydrology, Lancaster Environment Centre, Library Avenue, Bailrigg, Lancaster, LA1 4AP, UK

* **Corresponding author email**: [staffan.roos@slu.se](mailto:staffan.roos@slu.se)

**Table SI 1.** The number of Kestrels found in different UK counties that were analysed for SGARs between 1997 and 2012.

| **Country and county** | **Year** | | | | | | | | | | | | | | | | |
| --- | --- | --- | --- | --- | --- | --- | --- | --- | --- | --- | --- | --- | --- | --- | --- | --- | --- |
|  | **1997** | **1998** | **1999** | **2000** | **2001** | **2002** | **2003** | **2004** | **2005** | **2006** | **2007** | **2008** | **2009** | **2010** | **2011** | **2012** | **Total** |
| **Scotland** |  |  |  |  |  |  |  |  |  |  |  |  |  |  |  |  |  |
| Aberdeenshire | 0 | 0 | 0 | 0 | 0 | 0 | 0 | 0 | 0 | 0 | 1 | 0 | 0 | 1 | 0 | 0 | 2 |
| Borders | 0 | 0 | 0 | 0 | 0 | 0 | 0 | 1 | 0 | 0 | 0 | 0 | 0 | 0 | 0 | 0 | 1 |
| Dumfries and Galloway | 0 | 0 | 0 | 0 | 0 | 0 | 0 | 1 | 0 | 0 | 0 | 0 | 0 | 0 | 0 | 0 | 1 |
| East Lothian | 0 | 0 | 0 | 0 | 0 | 0 | 1 | 0 | 0 | 0 | 0 | 0 | 0 | 0 | 0 | 0 | 1 |
| Fife | 0 | 0 | 0 | 0 | 0 | 0 | 0 | 0 | 0 | 0 | 1 | 0 | 1 | 0 | 0 | 0 | 2 |
| Grampian | 0 | 0 | 0 | 0 | 0 | 0 | 0 | 1 | 1 | 0 | 0 | 0 | 0 | 2 | 0 | 1 | 5 |
| Highland | 0 | 0 | 0 | 0 | 0 | 0 | 0 | 3 | 1 | 0 | 1 | 1 | 0 | 0 | 1 | 0 | 7 |
| Lothian | 0 | 0 | 0 | 0 | 0 | 0 | 1 | 0 | 0 | 0 | 0 | 0 | 0 | 0 | 0 | 0 | 1 |
| Orkney | 0 | 0 | 0 | 0 | 0 | 0 | 0 | 0 | 0 | 0 | 0 | 0 | 1 | 0 | 0 | 1 | 2 |
| Perth & Kinross | 0 | 0 | 0 | 0 | 1 | 0 | 0 | 0 | 0 | 0 | 0 | 0 | 0 | 0 | 0 | 0 | 1 |
| Stirling | 0 | 0 | 0 | 0 | 0 | 0 | 0 | 0 | 0 | 0 | 1 | 0 | 0 | 0 | 0 | 0 | 1 |
| Strathclyde | 0 | 0 | 0 | 0 | 0 | 0 | 1 | 0 | 0 | 1 | 0 | 0 | 2 | 0 | 1 | 0 | 5 |
| Tayside | 0 | 0 | 0 | 0 | 0 | 1 | 1 | 2 | 0 | 1 | 0 | 0 | 0 | 0 | 1 | 0 | 6 |
| **Scotland Total** | **0** | **0** | **0** | **0** | **1** | **1** | **4** | **8** | **2** | **2** | **4** | **1** | **4** | **3** | **3** | **2** | **35** |
|  |  |  |  |  |  |  |  |  |  |  |  |  |  |  |  |  |  |
| **England** |  |  |  |  |  |  |  |  |  |  |  |  |  |  |  |  |  |
| Berkshire | 0 | 0 | 0 | 0 | 0 | 0 | 0 | 1 | 0 | 0 | 0 | 0 | 0 | 0 | 0 | 2 | 3 |
| Buckinghamshire | 0 | 0 | 0 | 0 | 0 | 0 | 0 | 0 | 0 | 0 | 0 | 0 | 1 | 0 | 1 | 0 | 2 |
| Cambridgeshire | 1 | 1 | 0 | 4 | 2 | 3 | 4 | 3 | 8 | 4 | 0 | 4 | 1 | 1 | 1 | 1 | 38 |
| Cheshire | 0 | 0 | 0 | 1 | 0 | 0 | 0 | 1 | 1 | 0 | 0 | 0 | 0 | 1 | 1 | 0 | 5 |
| Cornwall | 0 | 0 | 0 | 0 | 0 | 0 | 1 | 0 | 0 | 0 | 0 | 0 | 0 | 0 | 0 | 0 | 1 |
| **England (continued)** |  |  |  |  |  |  |  |  |  |  |  |  |  |  |  |  |  |
| Cumbria | 0 | 0 | 0 | 0 | 0 | 2 | 0 | 0 | 0 | 0 | 0 | 0 | 1 | 0 | 0 | 0 | 3 |
| Derbyshire | 0 | 0 | 0 | 0 | 0 | 0 | 0 | 0 | 0 | 0 | 0 | 0 | 0 | 0 | 0 | 1 | 1 |
| Devon | 0 | 0 | 1 | 0 | 0 | 0 | 0 | 1 | 0 | 0 | 0 | 0 | 0 | 0 | 1 | 0 | 3 |
| Dorset | 0 | 0 | 0 | 1 | 0 | 0 | 0 | 0 | 0 | 0 | 0 | 0 | 0 | 0 | 0 | 1 | 2 |
| East Riding of Yorkshire | 0 | 0 | 0 | 0 | 0 | 2 | 0 | 0 | 0 | 0 | 0 | 0 | 2 | 0 | 0 | 0 | 4 |
| East Sussex | 0 | 0 | 0 | 0 | 0 | 1 | 0 | 0 | 0 | 0 | 0 | 0 | 0 | 0 | 0 | 0 | 1 |
| Essex | 1 | 0 | 1 | 0 | 0 | 0 | 0 | 2 | 0 | 1 | 0 | 0 | 1 | 0 | 0 | 0 | 6 |
| Gloucestershire | 0 | 0 | 0 | 0 | 0 | 0 | 1 | 0 | 0 | 0 | 0 | 0 | 0 | 0 | 0 | 0 | 1 |
| Hampshire | 0 | 0 | 0 | 0 | 0 | 2 | 1 | 1 | 0 | 2 | 0 | 0 | 0 | 1 | 2 | 3 | 12 |
| Hertfordshire | 0 | 0 | 0 | 0 | 0 | 0 | 0 | 1 | 0 | 2 | 0 | 0 | 0 | 0 | 0 | 0 | 3 |
| Isle of Wight | 0 | 0 | 0 | 0 | 0 | 0 | 0 | 0 | 0 | 0 | 0 | 1 | 0 | 0 | 0 | 0 | 1 |
| Kent | 0 | 0 | 0 | 0 | 1 | 1 | 0 | 0 | 1 | 0 | 0 | 0 | 0 | 0 | 2 | 1 | 6 |
| Lancashire | 0 | 0 | 0 | 0 | 0 | 0 | 0 | 0 | 0 | 0 | 0 | 1 | 0 | 0 | 0 | 0 | 1 |
| Leicestershire (with Rutland) | 0 | 0 | 0 | 0 | 0 | 0 | 0 | 1 | 1 | 0 | 1 | 0 | 1 | 0 | 0 | 0 | 4 |
| Lincolnshire | 0 | 0 | 1 | 0 | 1 | 2 | 0 | 2 | 0 | 0 | 0 | 1 | 0 | 0 | 2 | 0 | 9 |
| Norfolk | 0 | 2 | 2 | 3 | 1 | 1 | 3 | 6 | 1 | 7 | 0 | 0 | 0 | 0 | 0 | 0 | 26 |
| North Lincolnshire | 0 | 0 | 0 | 0 | 0 | 0 | 1 | 0 | 2 | 0 | 0 | 0 | 0 | 0 | 0 | 0 | 3 |
| Northamptonshire | 2 | 0 | 2 | 1 | 1 | 1 | 3 | 0 | 0 | 3 | 0 | 0 | 0 | 0 | 1 | 0 | 14 |
| Nottinghamshire | 0 | 0 | 0 | 0 | 0 | 0 | 0 | 0 | 1 | 0 | 0 | 0 | 0 | 0 | 0 | 0 | 1 |
| Oxfordshire | 0 | 0 | 0 | 0 | 0 | 0 | 0 | 0 | 1 | 2 | 0 | 0 | 1 | 0 | 1 | 0 | 5 |
| Shropshire | 2 | 0 | 0 | 1 | 0 | 0 | 0 | 0 | 1 | 0 | 0 | 0 | 0 | 0 | 0 | 0 | 4 |
| Somerset | 0 | 2 | 0 | 0 | 5 | 2 | 3 | 1 | 0 | 0 | 1 | 0 | 0 | 0 | 0 | 0 | 14 |
| **England (continued)** |  |  |  |  |  |  |  |  |  |  |  |  |  |  |  |  |  |
| Staffordshire | 0 | 0 | 0 | 0 | 0 | 0 | 0 | 2 | 0 | 0 | 0 | 0 | 0 | 0 | 0 | 0 | 2 |
| Suffolk | 1 | 0 | 1 | 0 | 0 | 0 | 0 | 4 | 0 | 4 | 1 | 0 | 0 | 0 | 0 | 0 | 11 |
| Surrey | 0 | 0 | 0 | 0 | 0 | 1 | 0 | 0 | 0 | 0 | 0 | 0 | 0 | 0 | 0 | 0 | 1 |
| West Sussex | 0 | 0 | 0 | 0 | 1 | 1 | 1 | 2 | 0 | 1 | 0 | 0 | 0 | 0 | 0 | 0 | 6 |
| West Yorkshire | 0 | 0 | 0 | 0 | 0 | 0 | 0 | 1 | 0 | 0 | 1 | 0 | 1 | 0 | 0 | 0 | 3 |
| Wiltshire | 0 | 0 | 0 | 0 | 0 | 0 | 0 | 1 | 1 | 0 | 0 | 0 | 0 | 0 | 1 | 0 | 3 |
| Worcestershire | 0 | 0 | 0 | 0 | 0 | 0 | 1 | 0 | 0 | 0 | 1 | 0 | 0 | 0 | 0 | 0 | 2 |
| **England Total** | **7** | **5** | **8** | **11** | **12** | **19** | **19** | **30** | **18** | **26** | **5** | **7** | **9** | **3** | **13** | **9** | **201** |
|  |  |  |  |  |  |  |  |  |  |  |  |  |  |  |  |  |  |
| **Unknown** | **0** | **0** | **0** | **0** | **1** | **0** | **0** | **1** | **0** | **0** | **0** | **1** | **0** | **0** | **0** | **1** | **4** |
|  |  |  |  |  |  |  |  |  |  |  |  |  |  |  |  |  |  |
| **Wales** |  |  |  |  |  |  |  |  |  |  |  |  |  |  |  |  |  |
| Carmarthenshire | 0 | 0 | 0 | 0 | 0 | 0 | 0 | 1 | 0 | 0 | 0 | 0 | 0 | 0 | 0 | 0 | 1 |
| **Wales Total** | **0** | **0** | **0** | **0** | **0** | **0** | **0** | **1** | **0** | **0** | **0** | **0** | **0** | **0** | **0** | **0** | **1** |
| **TOTAL** | **7** | **5** | **8** | **11** | **14** | **20** | **23** | **40** | **20** | **28** | **9** | **9** | **13** | **6** | **16** | **12** | **241** |

**Table SI 2.** The number of Kestrels tested, the mean (± SE) concentration (μg/ g wet liver weight) of the three most commonly used SGARs and the mean total SGAR concentration in livers of Kestrel found in different counties across the UK. In addition, the mean number of SGAR compounds per Kestrel and percentage Kestrels with detectable levels of any SGAR are shown. The level of detection (LoD) from the modern and more sensitive liquid chromatography mass spectrometry (LCMS) technique was adjusted so that SGAR values of < 0.025 μg/ g wet liver weight were treated as 0. The mean concentrations are from birds with detectable levels of individual SGARs only. Blanks means that no Kestrels in that county had detectable levels of the specific SGAR.

| **Country and county** | **N** | **Brodifacoum** | **Difenacoum** | **Bromadiolone** | **Total SGAR** | **Number of SGARs** | **% with SGAR** |
| --- | --- | --- | --- | --- | --- | --- | --- |
| **Scotland** |  |  |  |  |  |  |  |
| Aberdeenshire | 2 |  |  |  |  | 0.000 ± 0.000 | 0.00 |
| Borders | 1 |  |  |  |  | 0.000 ± 0.000 | 0.00 |
| Dumfries and Galloway | 1 |  | 0.111 ± 0.000 |  | 0.111 ± 0.000 | 1.000 ± 0.000 | 100.00 |
| East Lothian | 1 |  | 0.07 ± 0.000 |  | 0.070 ± 0.000 | 1.000 ± 0.000 | 100.00 |
| Fife | 2 |  |  | 0.727 ± 0.000 | 0.727 ± 0.000 | 0.500 ± 0.500 | 50.00 |
| Grampian | 5 |  |  |  |  | 0.000 ± 0.000 | 0.00 |
| Highland | 7 |  |  | 0.138 ± 0.008 | 0.138 ± 0.008 | 0.286 ± 0.184 | 28.57 |
| Lothian | 1 |  |  |  |  | 0.000 ± 0.000 | 0.00 |
| Orkney | 2 |  |  | 0.051 ± 0.000 | 0.051 ± 0.000 | 0.500 ± 0.500 | 50.00 |
| Perth & Kinross | 1 |  |  |  |  | 0.000 ± 0.000 | 0.00 |
| Stirling | 1 |  |  |  |  | 0.000 ± 0.000 | 0.00 |
| Strathclyde | 5 |  |  | 0.238 ± 0.165 | 0.238 ± 0.165 | 0.400 ± 0.245 | 40.00 |
| Tayside | 6 |  |  |  |  | 0.000 ± 0.000 | 0.00 |
| **Scotland Total** | **35** |  | **0.091 ± 0.021** | **0.255 ± 0.108** | **0.214 ± 0.083** | **0.229 ± 0.072** | **22.86** |
|  |  |  |  |  |  |  |  |
|  |  |  |  |  |  |  |  |
|  |  |  |  |  |  |  |  |
|  |  |  |  |  |  |  |  |
| **England** |  |  |  |  |  |  |  |
| Berkshire | 3 |  | 0.007 ± 0.000 | 0.183 ± 0.109 | 0.185 ± 0.108 | 1.333 ± 0.333 | 100.00 |
| Buckinghamshire | 2 |  |  | 0.173 ± 0.077 | 0.173 ± 0.077 | 1.000 ± 0.000 | 100.00 |
| Cambridgeshire | 38 | 0.057 ± 0.022 | 0.055 ± 0.020 | 0.167 ± 0.046 | 0.148 ± 0.032 | 0.895 ± 0.163 | 55.26 |
| Cheshire | 5 |  | 0.028 ± 0.020 | 0.198 ± 0.003 | 0.113 ± 0.050 | 0.800 ± 0.200 | 80.00 |
| Cornwall | 1 |  |  |  |  | 0.000 ± 0.000 | 0.00 |
| Cumbria | 3 |  |  | 0.120 ± 0.000 | 0.120 ± 0.000 | 0.333 ± 0.333 | 33.33 |
| Derbyshire | 1 |  | 0.091 ± 0.000 | 0.344 ± 0.000 | 0.434 ± 0.000 | 2.000 ± 0.000 | 100.00 |
| Devon | 3 |  | 0.060 ± 0.000 | 0.062 ± 0.000 | 0.061 ± 0.001 | 0.667 ± 0.333 | 66.67 |
| Dorset | 2 |  | 0.037 ± 0.000 | 0.165 ± 0.000 | 0.202 ± 0.000 | 1.000 ± 1.000 | 50.00 |
| East Riding of Yorkshire | 4 |  | 0.036 ± 0.016 | 0.784 ± 0.243 | 0.546 ± 0.281 | 1.000 ± 0.408 | 75.00 |
| East Sussex | 1 |  |  |  |  | 0.000 ± 0.000 | 0.00 |
| Essex | 6 |  | 0.581 ± 0.450 | 0.103 ± 0.032 | 0.394 ± 0.243 | 1.000 ± 0.365 | 66.67 |
| Gloucestershire | 1 |  |  |  |  | 0.000 ± 0.000 | 0.00 |
| Hampshire | 12 |  | 0.068 ± 0.046 | 0.277 ± 0.078 | 0.306 ± 0.072 | 0.833 ± 0.241 | 58.33 |
| Hertfordshire | 3 | 0.041 ± 0.010 | 0.081 ± 0.005 | 0.108 ± 0.023 | 0.230 ± 0.029 | 2.000 ± 1.000 | 66.67 |
| Isle of Wight | 1 |  |  |  |  | 0.000 ± 0.000 | 0.00 |
| Kent | 6 | 0.070 ± 0.000 | 0.070 ± 0.036 | 0.542 ± 0.338 | 0.490 ± 0.322 | 1.333 ± 0.422 | 83.33 |
| Lancashire | 1 |  |  |  |  | 0.000 ± 0.000 | 0.00 |
| Leicestershire (with Rutland) | 4 |  | 0.159 ± 0.000 | 0.097 ± 0.030 | 0.150 ± 0.036 | 1.000 ± 0.408 | 75.00 |
| Lincolnshire | 9 | 0.157 ± 0.081 | 0.083 ± 0.054 | 0.454 ± 0.125 | 0.509 ± 0.132 | 1.778 ± 0.324 | 88.89 |
| Norfolk | 26 | 0.140 ± 0.068 | 0.136 ± 0.064 | 0.176 ± 0.044 | 0.277 ± 0.076 | 1.269 ± 0.197 | 69.23 |
| North Lincolnshire | 3 |  | 0.030 ± 0.000 | 0.246 ± 0.000 | 0.276 ± 0.000 | 0.667 ± 0.667 | 33.33 |
| Northamptonshire | 14 |  | 0.128 ± 0.050 | 0.209 ± 0.082 | 0.202 ± 0.074 | 0.857 ± 0.177 | 71.43 |
| Nottinghamshire | 1 |  |  |  |  | 0.000 ± 0.000 | 0.00 |
| Oxfordshire | 5 |  | 0.210 ± 0.062 | 0.056 ± 0.027 | 0.195 ± 0.052 | 1.000 ± 0.447 | 60.00 |
| Shropshire | 4 | 0.093 ± 0.000 | 0.320 ± 0.000 | 0.179 ± 0.000 | 0.197 ± 0.066 | 0.750 ± 0.250 | 75.00 |
| Somerset | 14 | 0.095 ± 0.030 | 0.088 ± 0.021 | 0.189 ± 0.068 | 0.205 ± 0.042 | 1.000 ± 0.257 | 64.29 |
| Staffordshire | 2 | 0.530 ± 0.000 | 0.003 ± 0.000 |  | 0.267 ± 0.264 | 1.000 ± 0.000 | 100.00 |
| Suffolk | 11 | 0.196 ± 0.000 | 0.286 ± 0.129 | 0.263 ± 0.230 | 0.337 ± 0.131 | 0.909 ± 0.211 | 72.73 |
| Surrey | 1 |  | 0.009 ± 0.000 |  | 0.009 ± 0.000 | 1.000 ± 0.000 | 100.00 |
| West Sussex | 6 | 0.034 ± 0.023 | 0.326 ± 0.000 | 0.148 ± 0.061 | 0.197 ± 0.052 | 1.167 ± 0.307 | 83.33 |
| West Yorkshire | 3 | 0.577 ± 0.000 | 0.289 ± 0.258 | 0.091 ± 0.000 | 0.622 ± 0.591 | 1.333 ± 0.882 | 66.67 |
| Wiltshire | 3 | 1.041 ± 0.000 | 0.004 ± 0.000 | 0.269 ± 0.225 | 0.617 ± 0.571 | 1.667 ± 0.333 | 100.00 |
| Worcestershire | 2 | 0.026 ± 0.000 | 0.129 ± 0.000 | 0.575 ± 0.535 | 0.653 ± 0.613 | 2.000 ± 1.000 | 100.00 |
| **England Total** | **201** | **0.168 ± 0.049** | **0.121 ± 0.021** | **0.238 ± 0.028** | **0.276 ± 0.029** | **1.030 ± 0.065** | **66.67** |
|  |  |  |  |  |  |  |  |
| **Unknown** | 4 | 1.531 ± 0.000 | 0.125 ± 0.000 | 0.203 ± 0.000 | 0.930 ± 0.602 | 0.750 ± 0.479 | 50.00 |
|  |  |  |  |  |  |  |  |
| **Wales** |  |  |  |  |  |  |  |
| Carmarthenshire | 1 |  |  |  |  | 0.000 ± 0.000 | 0.00 |
| **Wales Total** | **1** |  |  |  |  | **0.000 ± 0.000** | **0.00** |
| **TOTAL** | **241** | **0.222 ± 0.072** | **0.121 ± 0.020** | **0.238 ± 0.027** | **0.282 ± 0.029** | **0.905 ± 0.058** | **59.8** |

a)

**
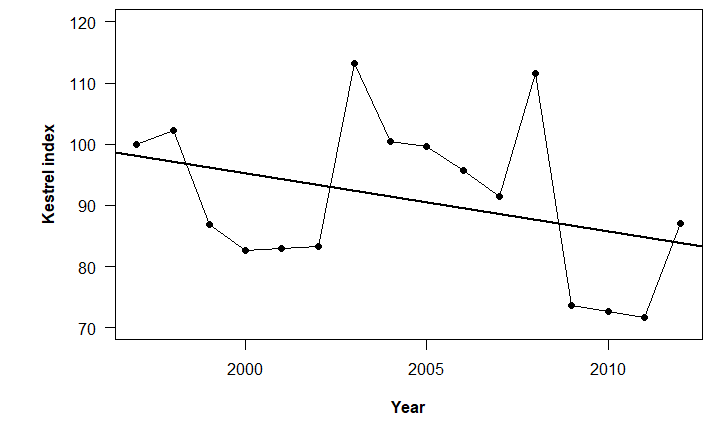
**

b)

**
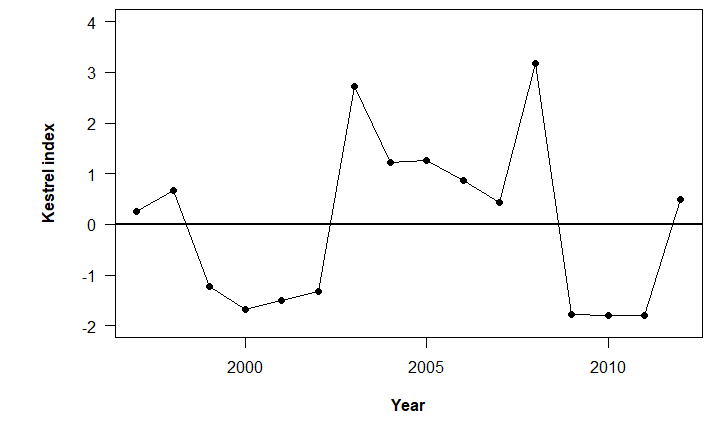
**

**Figure S1.** In a) the unadjusted and in b) the detrended time series of the Kestrel Breeding Bird Survey population index between 1997 and 2012. Before detrending the data, the population index data were transformed to achieve normal distribution. The black filled circles show the annual population index estimate. The straight solid lines are the regression lines.
